# Modulation of Innate Immune Mechanisms to Enhance *Leishmania* Vaccine-Induced Immunity: Role of Coinhibitory Molecules

**DOI:** 10.3389/fimmu.2016.00187

**Published:** 2016-05-13

**Authors:** Sreenivas Gannavaram, Parna Bhattacharya, Nevien Ismail, Amit Kaul, Rakesh Singh, Hira L. Nakhasi

**Affiliations:** ^1^Laboratory of Emerging Pathogens, Division of Emerging and Transfusion Transmitted Diseases, Food and Drug Administration, Silver Spring, MD, USA; ^2^Department of Biochemistry, Banaras Hindu University, Varanasi, India

**Keywords:** leishmaniasis, vaccine, coinhibitory molecules, CD200R, PD-L1, CTLA-4, TIM-3, CD200

## Abstract

No licensed human vaccines are currently available against any parasitic disease including leishmaniasis. Several antileishmanial vaccine formulations have been tested in various animal models, including genetically modified live-attenuated parasite vaccines. Experimental infection studies have shown that *Leishmania* parasites utilize a broad range of strategies to undermine effector properties of host phagocytic cells, i.e., dendritic cells (DCs) and macrophages (MΦ). Furthermore, *Leishmania* parasites have evolved strategies to actively inhibit T_H_1 polarizing functions of DCs and to condition the infected MΦ toward anti-inflammatory/alternative/M2 phenotype. The altered phenotype of phagocytic cells is characterized by decreased production of antimicrobial reactive oxygen, nitrogen molecules, and pro-inflammatory cytokines, such as IFN-γ, IL-12, and TNF-α. These early events limit the activation of T_H_1-effector cells and set the stage for pathogenesis. Furthermore, this early control of innate immunity by the virulent parasites results in substantial alteration in the adaptive immunity characterized by reduced proliferation of CD4^+^ and CD8^+^ T cells and T_H_2-biased immunity that results in production of anti-inflammatory cytokines, such as TGF-β, and IL-10. More recent studies have also documented the induction of coinhibitory ligands, such as CTLA-4, PD-L1, CD200, and Tim-3, that induce exhaustion and/or non-proliferation in antigen-experienced T cells. Most of these studies focus on viral infections in chronic phase, thus limiting the direct application of these results to parasitic infections and much less to parasitic vaccines. However, these studies suggest that vaccine-induced protective immunity can be modulated using strategies that enhance the costimulation that might reduce the threshold necessary for T cell activation and conversely by strategies that reduce or block inhibitory molecules, such as PD-L1 and CD200. In this review, we will focus on the polarization of antigen-presenting cells and subsequent role of costimulatory and coinhibitory molecules in mediating vaccine-induced immunity using live-attenuated *Leishmania* parasites as specific examples.

## Introduction

Visceral leishmaniasis (VL) has an annual incidence of 0.2–0.4 million cases globally and results in about 60,000 deaths (1). It has been reported that 0.7–1.2 million cases of cutaneous leishmaniasis (CL) occur around the globe (2). The disease currently affects 12 million people with 350 million people at risk of infection. The majority of patients with CL or VL develop a long-term protective immunity after cure from infection, which suggests that development of an effective vaccine against leishmaniasis is possible (3, 4). High frequency of asymptomatic infections, toxicity of currently available drugs, and increasing parasite resistance underline the need for an effective prophylactic vaccine against leishmaniasis (5).

Extensive studies in murine models that acquire resistance to *Leishmania major* infection have resulted in a broader understating of the mediators of protection, primarily a T_H_1-biased response (6). However, the principal determinants for inducing a strong T_H_1-type response following infection with virulent *Leishmania* parasites including expression of MHC-I/II, CD40, CD80/CD86, and cytokines, such as IL-12, by the antigen-presenting cells (APCs) [dendritic cells (DCs) and macrophages (MΦ)] are targets for alteration by the parasites (7, 8). *Leishmania* parasites can survive in a wide range of cell types. The parasites are phagocytosed by neutrophils first and are taken up by the MΦ and DCs without causing an overt immunological reaction (6). The multifarious interactions between *Leishmania* and the host APCs have profound effects on the final outcome of the interaction, either host resistance or susceptibility. MΦ are not only the primary host cell for *Leishmania* that permit parasite proliferation but also the major effector cells in eliminating the infection. The effective clearance of parasites by MΦ depends on the activation of an appropriate immune response usually initiated by the DCs (8).

## Reprograming of Macrophage/DC Differentiation

*Leishmania donovani* resides predominantly in host MΦ where it enters by phagocytosis and establishes itself within parasitophorous vacuoles (9). Macrophage responses to parasites lead to discrete, stereotyped phenotypes, which are usually a combination of inflammatory and anti-inflammatory functions (8). This plasticity in macrophage function has been defined either as classically activated (M1 phenotype) representing leishmanicidal activity or an alternatively activated state (M2 phenotype) that confers susceptibility to infection (10).

Classically activated macrophages (CAM) are primed by T_H_1 (or pro-inflammatory) cytokines and triggered by microbial products to produce antimicrobial molecules, such as reactive oxygen species (ROS) and nitric oxide (NO), through the action of inducible nitric oxide synthase (iNOS) and subsequently acquire a heightened effector function (11–13). CAM activation is also characterized by the induction of an array of pro-inflammatory cytokines, such as tumor necrosis factor [TNFα, IL-1β, and interleukin (IL)-12], which amplify T_H_1 immune responses (14–16). Specifically, IL-12 is a pivotal cytokine required for CD4^+^ T_H_1 development and production of IFN-γ (17). Since CAMs acquire properties necessary for the destruction of invading pathogens and priming the innate immune response, *Leishmania* parasites have evolved mechanisms to subvert microbicidal functions of the CAMs through depletion of antimicrobial molecules, such as ROS and NO (18–20), thereby reprograming the infected MΦ to an alternative activation state (21). Alternative macrophage activation is mainly induced by T_H_2 cytokines (22, 23) that antagonize the microbicidal properties of CAMs (8).

The enhanced T_H_2 response during virulent *Leishmania* infection leads to enhanced arginase activities in the MΦ, a prototypic alternative activation marker in mouse MΦ that allows parasite survival (22–24). Of the T_H_2 cytokines, IL-10 has emerged as the principal cytokine responsible for disease pathogenesis (25). IL-10 induced during *Leishmania* infection inhibits microbicidal activity of MΦ by attenuating the generation of NO and pro-inflammatory cytokines (26). Therefore, reprograming of the MΦ enables *Leishmania* parasites to evade the antimicrobial innate immune response and to proliferate within the phagolysosome of the macrophage.

The critical balance between host-protective T_H_1 (or pro-inflammatory) versus disease-promoting T_H_2 (or anti-inflammatory) effector responses determines the outcome of infection in leishmaniasis. This outcome is dictated by the relative levels of IL-12 and IL-10 produced by the classical/M1 and alternative/M2 MΦ, respectively (8, 16, 21). Previous studies have also shown that the MΦ are able to regulate immunologic outcomes on their own *via* directing the T cell response (27, 28). For example, infection of MΦ with virulent *Leishmania* induces a parasite-favoring T_H_2 response instead of a host-protective T_H_1 response (29). Interestingly, studies have shown that classical/pro-inflammatory/M1 MΦ have a direct role in the induction of T_H_1-polarized response (16, 27, 28, 30). Such M1 macrophage-induced T cell polarization was further linked with an induction of protection in several vaccine studies including recombinant BCG, attenuated *West Nile virus* (WNV), and live-attenuated measles virus (31–33). These recent studies demonstrated that the vaccine antigens, indeed, reprogram the MΦ to induce a pro-inflammatory response and result in improved protective immunity. Likewise, our studies with genetically modified live-attenuated *L. donovani* parasites revealed that these attenuated parasites induce classical activation of MΦ that direct host-protective T_H_1 response in mice (21). A recent study has also reported that M2 polarization of monocytes–macrophages is a hallmark of post kala-azar dermal leishmaniasis that sustains chronic lesions (10). Additionally, repolarization of the monocytes to M1 type by antileishmanial drugs as stated above suggests that switching from M2 to M1 phenotype might also be important in a therapeutic setting similar to what our studies indicated with prophylactic vaccination (21).

Although an effective clearance of parasites involves antimicrobial activity of the MΦ, the main host cell for *Leishmania* parasites, it also requires the activation of an appropriate immune response that is initiated by DCs (7, 8). Since MΦ are not the major producers of IL-12, potentiation of adaptive immune response depends primarily on the DC-derived IL-12 (34). In addition, parasite-infected MΦ are incompetent in priming naive CD4^+^ T cells as well as stimulating antigen (Ag)-specific CD4^+^ T cells (35). Thus, DCs play a crucial role in coordinating immune responses in leishmaniasis by providing the IL-12 necessary for the induction of protective T_H_1 response (36, 37). In both *L. major* and *L. donovani* mouse studies, the central role of DCs in determining the T_H_1/T_H_2 balance as well as the outcome of disease is demonstrated (36, 38). Specifically, *L. donovani* infection is shown to cause DCs to produce IL-12 that leads to NK cell activation (36). In the spleen, interaction between DCs and T cells occurs in the periarteriolar lymphoid sheath (PALS) into which DC and T cells migrate from the marginal zone (MZ) along chemokine gradients, especially CCL19/21, and this interaction between DCs and the T cells is necessary to produce protective immunity against *L. donovani* (39). Leon et al. also reported that monocyte–DCs formed at the infection site control the induction of protective T_H_1 responses against *Leishmania* (37). Nevertheless, infection with virulent *Leishmania* parasites is shown to prevent development of protective T_H_1 immunity by dysregulating DC function (7, 40). Additionally, Moll et al. reported that *L. major* parasites inhibit T_H_1 polarizing functions of epidermal-derived DCs (Langerhans cells) by upregulating IL-4 receptor expression along with concomitant downregulation of IL-12p40 production (41). Of interest, virulent *Leishmania* interferes with intracellular signaling in DCs, which affects their antigen-presenting functions, and hence, their ability to induce optimal cell-mediated immunity against the parasite (7, 42). DCs infected with intracellular pathogens, such as *Leishmania*, have been shown to elicit an MHC class-I-dependent CD8^+^ T-cell response, a process referred to as cross-presentation (43). Further studies have demonstrated the role of *Leishmania*-induced cleavage of proteins, such as SNAREs, that mediate the fusion of phagosomes with lysosomes, a key process in the antigen processing and presentation to the T cells (44, 45).

The role of DCs in priming a protective T_H_1 response is also illustrated by DC vaccination studies where exogenous administration of antigen-loaded DCs showed promising results in the treatment of different forms of leishmaniasis (46, 47). Curiously, adoptive transfer of DCs pulsed *ex vivo* with soluble *L. donovani* Ags (SLDA) to naive mice induced the Ag-specific production of IFN-γ and increased the percentage of activation markers on spleen lymphocytes. Moreover, SLDA-pulsed DCs engineered by retroviral gene transfer techniques to secrete high levels of biologically active murine-IL-12 augmented this immune response, further indicating the central role of DC secreted IL-12 in potentiating a protective response (48). Additionally, Schnitzer et al. have reported that DCs pulsed *ex vivo* with *L. major* antigen-induced protection in otherwise susceptible mice against subsequent challenges with the parasites (49). While it is well documented that antigen-loaded DC-based vaccines can induce protective immunity against *Leishmania* pathogenesis (50), live-attenuated parasites might be more practical in inducing protective immunity by modulating the DC function as illustrated by studies performed using DC-based vaccines. Taken together, these studies indicate that for an antileishmanial vaccine to induce protective immunity, T_H_1 favoring conditions in the DCs is a necessary requirement and live-attenuated parasite vaccines might be able to set in those conditions.

## Determinants of T Cell Immunity

An effective long-term protection requires activation of adaptive immunity mediated by T lymphocytes. APCs, primarily DCs, present processed antigens in combination with major histocompatibility complex to naive T cells displaying the corresponding T cell receptor. However, this presentation also requires additional signals arising from positive and negative coreceptors, i.e., costimulatory and coinhibitory molecules. The response of the T cell thus activated requires an amalgam of signals from the immunological synapse, and T cell activation can only occur when the stimulatory/inhibitory signals are able to overcome a certain threshold (51). Of the stimulatory signals, CD28 is the best-studied costimulatory molecule. Involvement of CD28 in the immunological synapse decreases the amount of antigen necessary to elicit T cell activation. Importantly, inflammatory signals regulate expression of CD28-binding partners, such as B7-1 (CD80) and B7-2 (CD86). Previous studies have identified suppression of costimulatory signals in DCs infected with virulent *Leishmania* parasites that results in poor IL-12 production and CD4^+^ T cell priming (36, 52–54). A T_H_2-polarized response was observed in CD40^−/−^ mice as indicated by excessive IL-4 and low IFN-γ. A direct role for CD40:CD40L ligation was shown not only in production of IL-12p70 but also in activation of T cells by the DCs (55, 56). Deficient expression of costimulatory CD80 was observed in *L. donovani*-infected MΦ (57). Similarly, CD86 has been shown to orchestrate either a T_H_1 or T_H_2 type response depending on the relative contribution of CD80 (58). More recently, several immune inhibitory mechanisms have been explored, which control exacerbated immune response of the host to prevent self-damage from unchecked inflammation (59). These mechanisms not only control the effector function of immune cells but also reprogram them for their alternate functions, such as humoral immunity and tissue remodeling, to maintain homeostasis between an immune response and immune tolerance. The interplay between signals arising from costimulatory molecules and coinhibitory molecules has been identified as a critical determinant in T cell activation. It has been hypothesized that costimulatory signals may act like a “rheostat” to modulate T cell activation in that costimulatory molecules reduce the TCR signaling threshold necessary for T cell activation, whereas inhibitory molecules restrict T cell activation (60).

Although there is considerable debate over the requirements for maintaining protection against reinfection in *Leishmania*, studies have shown that antigen-specific memory T cells are a principal component of protective immunity to intracellular pathogens, such as *L. major* (61–65). The memory T cells are distinguished by their ability (i) to survive long term in secondary lymphoid tissues and (ii) to undergo rapid and robust proliferation upon reinfection and acquisition of effector function. Yet, our understanding of T cell differentiation and memory formation is mainly derived from models of acute viral and bacterial infections, such as *Lymphocytic Choriomeningitis Virus*, *Vaccinia virus*, and *Listeria monocytogenes*. Persistent infections, such as *Leishmania*, may differ in significant ways from these models in the T cell response dynamics. Several dysfunctions including severe limitation in T cell expansion, delay in peak T cell expansion, anergy, and expression of exhaustion markers in chronic infections have been reported (66–69).

**Figure 1:**
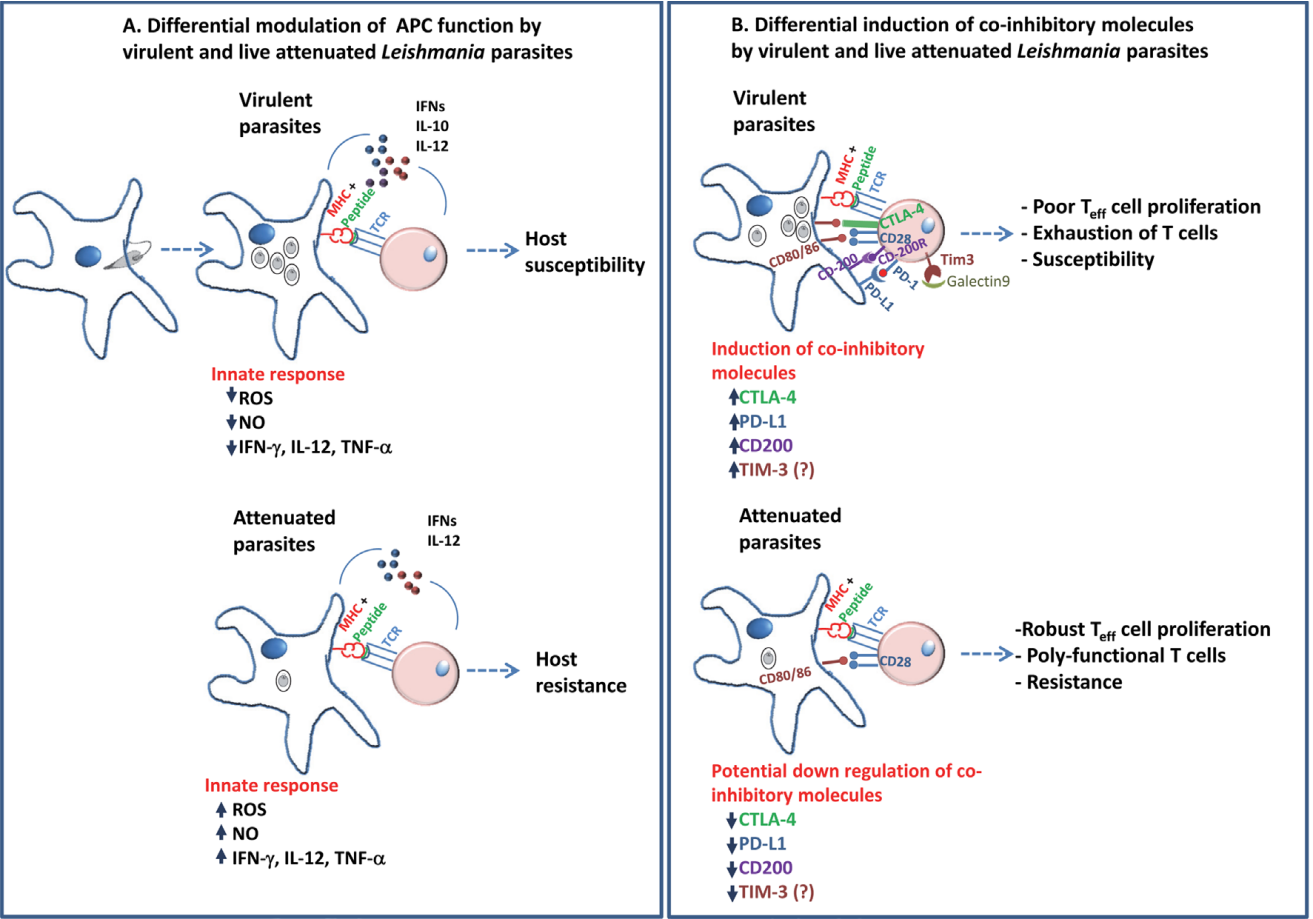
**A schematic diagram describing the differential modulation of antigen-presenting cells in the early innate response and role of coinhibitory molecules in adaptive immunity in *Leishmania* pathogenesis is shown**. **(A)** Virulent *Leishmania donovani* parasites are known to suppress antigen-presenting activity of DCs and macrophages. This is shown to be accompanied by reduction in the inflammatory cytokines as compared to infection with live-attenuated *L. donovani* parasites. These differences in the early interaction with the host APCs by the parasites determine the outcome, i.e., host susceptibility in case of virulent parasites or host resistance in case of attenuated parasites. **(B)** Previous studies have shown the role of coinhibitory molecules, such as CTLA-4, PD-L1, and CD200, in facilitating the parasite survival by inducing a restrained pro-inflammatory (T_H_1) environment. The role of Tim-3 in *Leishmania* pathogenesis remains to be explored. The role of coinhibitory molecules in shaping the T cell immunity suggests that attenuated parasites might downregulate these pathways compared to virulent parasites in order to induce protective immunity. Blockade of the coinhibitory signals during priming by use of adjuvants might show potential improvement in the vaccine-induced immunity.

In an acute infection, the T cell response typically follows three phases: expansion, contraction, and memory. During the first phase, upon presentation of antigens by DCs, naive T cells divide and differentiate into effector cells that acquire the ability to produce the pro-inflammatory cytokines IFN-γ and TNF, as well as cytotoxic proteins such as granzymes and perforin. This cascade of events by which T lymphocytes undergo differentiation and clonal expansion is regulated by signaling *via* antigens, costimulation, and cytokine receptors that induce the expression of transcription factors that dictate the fate of the T cells to acquire either an effector function or memory precursors (70). In case of CD8^+^ T cells, the cell fates are controlled by a coordinated set of changes in the expression of the transcription factors Id2, T-bet, and Blimp-1, which promote terminally differentiated effector cells, and Foxo1, TCF-1, Eomes and Bcl-6, which promote development of memory precursors (71–73). Earlier studies in *L. donovani* demonstrated the role of CD8^+^ T cells in the resolution of infection (74). In virulent *L. donovani* infection of mice, induction of exhausted CD8^+^ T cells has been demonstrated (66). More recent studies have shown a role for the transcription factor IRF-5 in regulating the antigen-specific CD8^+^ T cells responses during murine *L. donovani* infection (75). Inflammatory response generated by IRF-5 is shown to induce the expression of HIF-α in DCs and to limit CD8^+^ T cell expansion (75). Development of CD4^+^ T cells responses following either a chronic infection in comparison are less well studied (76). However, the dynamics of T cell effector/memory responses in a prophylactic vaccine setting are less well understood. It may be argued that in contrast to virulent parasites, prophylactic vaccines are composed of attenuated parasites due to their inherent immunomodulatory attributes may be able to induce conditions optimal for the immune system to generate memory cells that confer protection against subsequent infection (Figure 1). On the other hand, recombinant antigens may, and often, require adjuvants to enhance T cell responses. More systematic studies with prophylactic candidate vaccines are necessary to reveal the necessary conditions that must precede a strong protective response.

## Coinhibitory Molecules in Pathogenesis and Vaccine Immunity

In a chronic infection, it is essential that the host immune response be appropriately controlled to respond to and remove pathogens while avoiding excessive production of inflammatory cytokines and chemical mediators, such as ROS. The immune inhibitory signals, therefore, can dampen the effects of excessive immune reactions, which can lead to increased tissue damage and morbidity and mortality. In the following sections, we describe the role of various coinhibitory molecules and discuss their potential roles in inducing protective immunity with implication to live-attenuated vaccines.

## CTLA-4

B7 molecules expressed by DCs and MΦ are upregulated following activation. These B7 molecules B7-1 (CD80) and B7-2 (CD86) have dual specificity for receptors CD28 and CTLA-4 on T cells (77). CTLA-4 is an inhibitory coreceptor that is induced rapidly by activated T cells (78). Because of its higher affinity to CD80/86 than CD28, CTLA-4 often outcompetes and excludes CD28 from the immunological synapse, thereby causing suppression of T cell activation. In symptomatic VL, both the splenic aspirates and PBMCs from *L. donovani*-infected humans showed higher level of CTLA-4 expression (69). Higher CTLA-4 expression was also observed in PKDL lesions suggesting an association with persistence of parasites (79). Higher frequencies of CTLA-4^+^ CD4^+^ T cells have been identified in HIV-*Leishmania* coinfections that are associated with a poor immunological profile that might explain the persistence and relapse of the *Leishmania* infection (80). However, treatment with anti-CTLA-4 has shown mixed results. Blockade of CTLA-4 resulted in enhanced granuloma maturation indicating parasite-killing activity in the liver; however, in splenic aspirate cultures of human VL samples, CTLA-4 blockade did not show an increase in IFN-γ production (69, 81). The role of CTLA-4 in T cell activation has not been studied in a *Leishmania* vaccine setting. Reports have shown that preventing CTLA-4 interactions can improve T cell activation in other vaccine models. For example, *in vivo* blockade of CTLA-4 enhances antigen-specific CD4^+^ T cell responses after peptide immunization in complete Freund’s adjuvant for cryptococcal infection (82). Transient CTLA-4 blockade increased the number of memory CD8^+^ T cells during low-dose *Listeria* infection in mice, and CTLA-4 blockade enhanced the response of memory CD8^+^ T cells (83). The mechanism by which blockade of CTLA-4 might exert its functions is not completely understood. It has been proposed that blocking CTLA-4 might dampen the IL-10-mediated suppressive effects since CTLA-4 is very highly expressed on Treg cells (84). CTLA-4 plays an important role in the homeostasis and function of a population of suppressive activities of Treg cells (85). A direct role for CTLA-4 in T cell activation is suggested by studies that showed removal of B7 molecules from the surface of APCs by blocking CTLA-4 and, thus, increase the signal threshold necessary for T cell activation (86). Taken together, these studies suggest that CTLA-4 might be a potential target for modulation by prophylactic vaccines because of its effects first on T cell activation by its effects on B7 molecules and second by its ability to affect Treg cells.

## Programed Death-1

Programed death-1 (PD-1) is an inhibitory receptor that is inducibly expressed on stimulated CD4^+^ T cells, CD8^+^ T cells, B cells, and monocytes (66, 68, 87). PD-1 modulates T cell receptors and CD28 signaling through recruitment of the phosphatase SHP2. PD-1 binds to B7 family ligands, PD-L1 (B7-H1/CD274) and PD-L2 (B7-DC/CD273) (88). PD-L1 expression occurs on a wide variety of cells, whereas PD-L2 expression is restricted to APCs (DCs, monocytes, and some B cell subsets). Expression of both PD-1 ligands is modulated by cytokines, such as IL-2, IL-7, IL-15, IL-21, and IFN-γ (87, 89). Expression of PD-1 is induced by antigen receptor ligation and is dampened after resolution of infection in the absence of TCR signaling (90). However, sustained PD-1 expression is reported in several chronic disease models, including leishmaniasis, malaria, and Chagas (91–95). Specific parasite molecules (LPG from *Leishmania mexicana*) have also been shown to induce PD-1 expression in CD8^+^ T cells and PD-L2 in MΦ (96). Sustained PD-1 expression is maintained primarily due to continuous TCR ligation, and PD-1 ligation dramatically shifts the dose-response curve, making T cells much less sensitive to T-cell receptor-generated signals (97). TCR signals promote demethylation of regulatory regions of the PD-l locus. Additionally, transcription factor T-bet binds upstream of the PD-1 gene and represses its transcription. Since T-bet expression is downregulated in persistent TCR stimulation, low T-bet expression enables PD-1 transcription (98). Elevated expression of T-bet and PD-1 in CD4^+^ T cells in the patients of tuberculosis was also reported (99). In addition, recent studies have also identified transcriptional factors, hypoxia-inducible factor (HIF)-1α and signal transducer and activation of transcription 3 (STAT-3), that act on the promoter of PD-L1 to regulate its expression. In addition, microRNAs, including miR-570, miR-513, miR-197, miR-34a, and miR-200, negatively regulate PD-L1 (100).

Sustained PD-1 expression is a hallmark of dysfunctional T cells and commonly found in chronic infections. Importantly, several studies have shown that interfering with the PD-1 pathway rescues function in exhausted T cells including *Leishmania* and *Plasmodium* infections (66, 68, 87, 93, 101). Similar observations were also described in hepatitis C virus-infected patients that interfering with the PD-1/PD-L1 pathway during the early stage of immune responses can result in improved T cell responses (102). PD-L1 blockade during acute herpes simplex virus (HSV)-1 infection is shown to increase the magnitude and polyfunctionality of the HSV-specific CD8^+^ effector responses (103).

An important role as a clinical intervention for the PD-1 pathway in T cell exhaustion was first shown in LCMV infection in mice (104) and later demonstrated to occur in a diverse range of clinically relevant chronic human infections, such as HIV infection, hepatitis B and hepatitis C virus infections, and cancer (105–107). Several studies also tested combination of PD-1 and other ligands in blockade therapies (108, 109). Compared to clinical advances in treating cancer, blockade of inhibitory receptors as a strategy to treat chronically infected patients has lagged behind.

In addition to the role of PD-L1/PD-1 in regulating T cell function, it has been proposed that PD-L1/PD-1 interaction through the regulation by IFN-γ might allow for recognition of minor epitopes that otherwise would not be selected. This is consistent with the observation that PD-1 engagement preferentially inhibits low-avidity antigen receptors and thus allows expansion of immunodominant clones (89). In addition, in the absence of PD-L1/PD-1 interactions, APCs provide stronger stimulation to T cells. This is supported by experiments showing a significant enhancement of HIV-specific T cell responses by blocking PD-L1 (110). As a result, blockade of the PD-1 pathway has more significant effects in promoting T cell activation during conditions of suboptimal antigen presentation, such as with low antigen dose or with weak or low numbers of APCs (89).

Taken together, these studies suggest that PD-1 pathway blockade is an attractive strategy to improve prophylactic vaccination by allowing for better antigen selection and better T cell functional responses. Even though PD-1 pathway blockade is an attractive strategy to improve prophylactic vaccination, few studies have focused on the PD-1 pathway during early stages of T cell responses. Most importantly, PD-1 blockade has been studied in assessing CD8^+^ T cell responses, whereas the role of the PD-1 pathway on CD4^+^ T cell differentiation, an important mediator of antileishmanial immunity, has been relatively neglected.

## CD200R

CD200R is an inhibitory receptor expressed in the cells of lymphoid lineage, such as NK cells, CD4^+^, and CD8^+^ T cells, especially upon stimulation (59). Its ligand, CD200 (OX2), is a glycoprotein expressed on a broad number of cell types, including solid tumors and hematologic malignancies. Studies have also shown differential expression of CD200R on T cell subsets in mice and humans including effector and central memory T cells (111).

CD200R1 is an Ig superfamily transmembrane glycoprotein expressed on the surface of myeloid cells; it can also be induced in certain T-cell subsets (112, 113). CD200R1 interacts with CD200, which is also an Ig superfamily transmembrane glycoprotein, to downregulate myeloid cell functions. CD200 is expressed on the surface of a variety of cells including neurons, epithelial cells, endothelial cells, fibroblasts, lymphoid cells, and astrocytes (112, 114–116). The regulation of CD200R1 signaling can occur by posttranslational modifications mainly by phosphorylation of tyrosine residues in the CD200R1 cytoplasmic tail or by the inducible expression or the downregulation of either CD200R1 or CD200. Each of these mechanisms can be exploited by pathogens.

Unlike most immune inhibitory receptors, CD200R1 does not contain an immunoreceptor tyrosine-based inhibitory motif (ITIM). Stimulation by CD200 leads to the phosphorylation of these tyrosines by Src kinases, which recruit the adapter protein downstream of tyrosine kinase (Dok2) through its PTB domain (117). Dok2 serves as the major initiator of signaling through CD200R1, beginning with binding to Ras-GTPase-activating protein (RasGAP) and is required for CD200R1 function (117). This is in contrast to ITIM containing inhibitory receptors, which utilize SHPs and SHIP-1 as the major initiator proteins and Dok proteins as secondary modulators of downstream signaling (117, 118).

Compared to other inhibitory molecules, CD200 is less well studied in parasitic infections. *Leishmania amazonensis*, which causes severe disease in both humans and mice, induces CD200 expression in bone marrow MΦ from wild-type mice (119). Induction of CD200 upon infection was essential for the parasite virulence and development of systemic Leishmaniasis. Addition of CD200-Fc restored the virulence of *L. amazonensis* in mice lacking CD200. Distinct differences in CD200 signaling have been identified with other *Leishmania* species. *L. major*, which causes only localized cutaneous lesions, does not induce CD200 in MΦ. Interestingly, however, treatment with CD200-Fc of *L. major*-infected mice caused the parasite to disseminate into a systemic infection similar to that of *L. amazonensis* (119). *L. amazonensis* has evolved to utilize CD200 expression as a mechanism for inhibiting both NO production and induction of iNOS during infection. Interestingly, *L. amazonensis* increased CD200 expression on MΦ. MΦ have generally been found to express CD200R1, which can then interact with non-myeloid cells expressing CD200. Interestingly, mice lacking CD200 induced robust antiviral immunity including virus-specific CD4^+^ T cell responses (120). In two chronic infection models including *Salmonella* and *Schistosoma*, upregulated CD200R expression has been shown to result in poor multifunctional CD4^+^ T cell responses (112). Upregulated CD200R expression was associated with the development of T_H_2-type response. These results indicate that CD200 could be a candidate target for investigation as a regulator of T cell immunity in prophylactic antileishmanial vaccines since multifunctional CD4^+^ T cells have been shown to be correlated with protection in several *Leishmania* vaccine studies (Figure 1) (121, 122).

## TIM-3

The Tim-3 protein is a member of the T cell immunoglobulin and mucin domain (Tim) family, which encompasses a group of type-I transmembrane proteins expressed by both innate and adaptive cell types within the immune system. All Tim proteins are expressed on the cell surface and have been shown to function as receptors for soluble or cell-associated ligands. Additionally, certain Tim proteins can be shed from the cell surface and be found in soluble forms, suggesting a role as cell-free ligands. To date, the IgV domain of Tim-3 has been shown to interact with phosphatidylserine (PS) displayed on the surface of apoptotic cells, the alarmin protein high mobility group box 1 (HMGB1), and Galectin-9, a widely expressed soluble protein with specificity for carbohydrate chains containing β-galactoside sugars. Of the several ligands, the interaction between Tim-3 and Galectin-9 has been shown to impact CD4^+^ T cell functions. Addition of Galectin-9 to cultured Tim-3 T_H_1-type CD4^+^ T (T_H_1) cells induced apoptosis and necrosis, while injection of Galectin-9 into mice blunted immune responses driven by antigen-specific T_H_1 cells. These studies indicated that binding of Galectin-9 to Tim-3 results in the suppression of T cell responses, which supports the notion that Tim-3 functions as an inhibitory receptor for T cells.

A role for Tim-3 in regulating responses by CD4^+^ T cells has been suggested based on studies where addition of Galectin-9 induced the death of Tim-3^+^ T_H_1 cells *in vitro*. Other studies using autoimmune disease models suggest that ligation of Tim-3 by Galectin-9 leads to suppression of T_H_1-dependent immune responses. Studies of microbial infections also sought to investigate how Tim-3 and Galectin-9 influence CD4^+^ T cell responses. Tim-3 overexpression was observed on T cells that were senescent and dysfunctional in HCV infection, and blockade of Tim-3 rescued dysfunctional CD4^+^ and CD8^+^ T cells (123). In contrast, active tuberculosis patients exhibited increased numbers of Tim-3-expressing CD4^+^ and CD8^+^ T cells, which preferentially displayed polarized effector memory phenotypes. Tim-3^+^ CD4^+^ and CD8^+^ T cell subsets showed greater effector functions for producing Th1/Th22 cytokines and CTL effector molecules than those lacking Tim-3 expression, and Tim-3^+^ T cells controlled intracellular Mtb replication in MΦ (124). Overall, these findings suggest that Tim-3 can promote CD4^+^ T cell responses mounted against Mtb infection. This conclusion contrasts with other studies that showed Tim-3 expression correlated with T cell exhaustion. Taken together, these findings raise the possibility that Tim-3 function is influenced by context and that Tim-3 may inhibit or promote CD4^+^ T cell responses depending upon the microbe involved and the characteristics of the immune response elicited by the infection. Elevated expression of Tim-3 expression on T cells from HIV-1-infected individuals correlated positively with HIV-1 viral load and CD38 expression and inversely with CD4^+^ T cell count. In progressive HIV-1 infection, Tim-3 expression was upregulated on HIV-1-specific CD8^+^ T cells (125). Blocking the Tim-3 signaling pathway restored proliferation and enhanced cytokine production in HIV-1-specific T cells (125). In addition, blocking of Tim-3 rescued macrophage and T cell function in HIV positive tuberculosis patients (126). Persistence of HCV was associated with lower frequencies of IL-21-producing CD4^+^ T cells, reduced proliferation, and increased expression of the inhibitory receptors Tim-3, PD-1, and CTLA-4 on HCV-specific CD8^+^ T cells. Progression to persistent infection was accompanied by increased plasma levels of the Tim-3 ligand Galectin-9 and expansion of Gal-9 expressing regulatory Treg cells (127). Thus, studies in a diverse pathogen models suggests a net negative impact of Tim-3 expression on T cell-dependent antiviral immune responses. However, studies in Mtb point out that the Tim-3 expression and T cell function may be pathogen-specific. Consistent with this, in murine malaria, expression of both Tim-3 and Galectin-9 were associated with liver damage and acute lung injury (128, 129), suggesting a role in pathogenesis. It would be of interest to study if Tim-3 signaling has any role in prophylactic parasite vaccines.

## Conclusion

Chronic infections with intracellular parasites have been shown to induce several inhibitory molecules that subvert the development of protective immunity in the host and favor the survival of the parasite by mainly preventing the development of functional T cell immunity. While the role of these coinhibitory signals in virulence is being explored, important questions regarding their roles in shaping the protective immunity in a prophylactic vaccine setting are being recognized. The role of inhibitor signals in not only regulating T cell functions but modifying the adaptive immunity suggests that these molecules could be potential targets for modulation by candidate vaccines. Importantly, studies using blockade of ligands, such as PD-L1 and CTLA-4, have revealed important insights as to the clinical importance of such interventions.

## Author Contributions

All authors listed have made substantial, direct, and intellectual contribution to the work and approved it for publication.

## Conflict of Interest Statement

The authors declare that the research was conducted in the absence of any commercial or financial relationships that could be construed as a potential conflict of interest.
